# Using urine to diagnose large‐scale mtDNA deletions in adult patients

**DOI:** 10.1002/acn3.51119

**Published:** 2020-07-07

**Authors:** Kristin N. Varhaug, Gonzalo S. Nido, Irenaeus de Coo, Pirjo Isohanni, Anu Suomalainen, Charalampos Tzoulis, Per Knappskog, Laurence A. Bindoff

**Affiliations:** ^1^ Department of Neurology Haukeland University Hospital Bergen Norway; ^2^ Department of Clinical Medicine (K1) University of Bergen Bergen Norway; ^3^ Neuro‐SysMed Department of Neurology Haukeland University Hospital Bergen Norway; ^4^ Department of Neurology Medical Spectrum Twente Enschede The Netherlands; ^5^ Department of Genetics and Cell Biology University of Maastricht Maastricht The Netherlands; ^6^ Research Programs Unit, Stem Cells and Metabolism Faculty of Medicine University of Helsinki Helsinki Finland; ^7^ Children´s Hospital University of Helsinki and Helsinki University Hospital Helsinki Finland; ^8^ HUSlab Helsinki University Hospital Helsinki Helsinki Finland; ^9^ Neuroscience Center University of Helsinki Hilife Helsinki Finland; ^10^ Center for Medical Genetics and Molecular Medicine Haukeland University Hospital Bergen Norway; ^11^ Department of Clinical Science University of Bergen Bergen Norway

## Abstract

**Objective:**

The aim of this study was to evaluate if urinary sediment cells offered a robust alternative to muscle biopsy for the diagnosis of single mtDNA deletions.

**Methods:**

Eleven adult patients with progressive external ophthalmoplegia and a known single mtDNA deletion were investigated. Urinary sediment cells were used to isolate DNA, which was then subjected to long‐range polymerase chain reaction. Where available, the patient`s muscle DNA was studied in parallel. Breakpoint and thus deletion size were identified using both Sanger sequencing and next generation sequencing. The level of heteroplasmy was determined using quantitative polymerase chain reaction.

**Results:**

We identified the deletion in urine in 9 of 11 cases giving a sensitivity of 80%. Breakpoints and deletion size were readily detectable in DNA extracted from urine. Mean heteroplasmy level in urine was 38% ± 26 (range 8 ‐ 84%), and 57% ± 28 (range 12 – 94%) in muscle. While the heteroplasmy level in urinary sediment cells differed from that in muscle, we did find a statistically significant correlation between these two levels (*R* = 0.714, *P* = 0.031(Pearson correlation)).

**Interpretation:**

Our findings suggest that urine can be used to screen patients suspected clinically of having a single mtDNA deletion. Based on our data, the use of urine could considerably reduce the need for muscle biopsy in this patient group.

## Introduction

In humans, mitochondria are the only extra‐nuclear organelles that have their own DNA; mitochondrial DNA (mtDNA). This 16.5 kb circular genome encodes 13 proteins that are subunits of respiratory chain complexes: the remaining protein subunits are encoded by genes within the nucleus. In addition to 13 proteins, the mtDNA encodes 22 tRNA and 2 rRNA that participate in mitochondrial translation. Multiple copies of mtDNA are present within each cell and a mutation in mitochondrial genome can therefore, affect some or all of the copies; coexistence of mutated and wild‐type mtDNA is known as heteroplasmy.

The first pathogenic mutations in mtDNA giving rise to human disease, were identified in 1988, and were single large‐scale mitochondrial deletions (single deletions).^1^ Subsequently more than 150 single‐nucleotide changes and rearrangements have been identified.^2^

Single deletions contribute to ~ 16% of all mtDNA mutations in adults.^3^ They are primarily sporadic events with a minimum prevalence of 1.5/100 000,^3^ although maternal transmission has been reported,^4^ and the risk of transmission is higher than initially assumed.^5^

Single deletions give rise to three classical phenotypes; Pearson disease, Kearns‐Sayre syndrome (KSS), and progressive external ophthalmoplegia (PEO). Pearson disease is a multisystem disorder of infancy, recognized usually by the presence of sideroblastic anemia.^6^ Patients who survive infancy develop KSS. KSS has been defined as onset of PEO before age 20, pigmentary retinopathy and at least one of the following features; cardiac conduction block, cerebellar ataxia, and/or cerebrospinal fluid protein concentration> 0.1 g/L.^6^ PEO is characterized by ptosis and limited eye movement, often accompanied by proximal weakness and myopathy, in addition to other nonmuscular symptoms like hearing loss, ataxia, and other neurological symptoms.^7^

A replicative selection against mutations in cells that retain the ability to divide makes the diagnosis of mtDNA disease challenging, since it usually means that blood sampling is considered unsuitable for identifying mtDNA mutations. This is particularly true for mtDNA deletions, where skeletal muscle is regarded as the tissue of choice for diagnosis. Recent research has, however, shown that urine sediment cells retain sufficiently high levels of mtDNA point mutations to allow successful diagnosis of common mutations such as the m.3243A> G mutation.^8^ These cells have also been used to identify single deletions and their breakpoints,^9^, ^10^ but the finding that urine mtDNA deletions levels were more representative of levels found in muscle in young patients raised questions whether these cells were appropriate for diagnosing adults with single deletions.^10^ There are, however, no studies that have addressed this question systematically. Our aim was therefore to investigate: (a) how robust urine was for detecting single deletions in urinary epithelial cells of adult patients, (b) if urine could be used to map and sequence deletion breakpoints, (c) if the degree of heteroplasmy detected in urine was similar to that in skeletal muscle biopsy and (d) if we could use our data to develop a clinical algorithm for investigating patients with PEO.

## Methods

### Patient samples

We investigated 11 patients from three different centres (Norway, Finland, The Netherlands).

In nine patients (pts 1‐5, 7‐10) urine and muscle samples were available while in two (pts 6 and 11) only urine samples were available. The size of the mtDNA deletion was, however, known from diagnostic studies of muscle. Where available, the patient`s muscle DNA was studied in parallel with urine DNA in all performed assays. The time from diagnosis in muscle to time of urinary sample varied from 0 up to 18 years.

Urine samples were collected independent of time of day, and immediately centrifuged for 15 min at 2000 g, the supernatant discarded and the pellet retained. The pellet was stored at −20°C before use. A volume of 200 ul of ultra‐pure water was added to the pellet, before DNA was extracted using the QIAmp DNA Mini‐kit (Qiagen). The manufacturer`s tissue protocol was used for extracting DNA from muscle, and for the urine samples the manufacturer`s protocol for body fluids was used.

### Polymerase chain reactions (PCR)

Two long‐range PCR reactions (L‐PCR) were used to amplify mtDNA across the major arc, generating either an ~ 8 kb or a ~ 16 kb product in wild‐type mtDNA. A control sample from a patient with a normal muscle biopsy was run together.

Following primers were used: 8F (8232 – 8263) 5´‐TAAAAATCTTTGAAATAGGGCCCGTATTTACC‐3´ and L8R (16496 – 16465) 5´ ‐ CGGATACAGTTCACTTTAGCTACCCCCAAGTG‐3´; 15F (1650‐1671) 5´‐AACTTAACTTGACCGCTCTGAG −3´ and 15R (019‐001) 5´‐GGGTGATAGACCTGTGATC‐3´.

The PCR products were run on a 0.7% agarose gel at 40 V for approximately 4 hours. The wells were loaded with 5 μL PCR product and 1 μL loading dye.

Primer sequences for “walking‐PCR” were L8R in combination with (5855‐5875) TGTAAAACGACGGCCAGTACCTCAATCACACTACTCC, (6863‐6882) TGTAAAACGACGGCCAGTATTTAGCTGACTCGCCACAC or (7713‐7723) TGTAAAACGACGGCCAGTTCCTAACACTCACAACAAAAC.

To establish that DNA extracted from urine sediment cells could be used to define the deletion breakpoint, we chose two of the patients for Sanger sequencing (pt 8 and pt 10). Standard PCR reactions encompassing the deletion breakpoints were performed, and a total of 25 μL with PCR‐product was run on a 0.7% agarose gel. The band was extracted and PCR‐product purified using the QIAquick Gel Extraction Kit (Qiagen).

PCR‐products were sequenced with BigDye® Terminator v3.1 (ThermoFisher Scientific).

### Quantitative polymerase chain reaction

We quantified the level of mtDNA deletion using quantitative PCR (qPCR). The mtDNA regions *ND1* and *ND4* were amplified together with the single‐copy nuclear gene *APP*. The following primer, probes and conditions were used. *MTND1*: forward primer: L3485‐3504: 5´‐CCCTAAAACCCGCCACATCT‐3´, reverse primer: H3553‐3532: 5´GAGCGATGGTGAGAGCTAAGGT‐3´, TaqMan MGB probe: L3506‐3529: FAM‐CCATCACCCTCTACATCACCGCCC. *MTND4*: forward primer: L12087‐12109: 5´‐CCATTCTCCTCCTATCCCTCAAC‐3´, reverse primer: H12200‐12170: 5`‐ CACAATCTGATGTTTTGGTTAAACTATATTT‐3´, TaqMan MGB probe: L12111‐12138: NED‐CCGACATCATTACCGGGTTTTCCTCTTG. *APP*: forward primer: 5´TGTGTGCTCTCCCAGGTCTA‐3´, reverse primer: 5´CAGTTCTGGATGGTCACTGG‐3´, TaqMan MGB probe: VIC – CCCTGAACTGCAGATCACCAATGTGGTAG. The reactions were run in triplicate with DNA template of ~ 10 ng/µl.

Thermal cycle: one cycle at 95º for 20 s, 45 cycles at 95º for 3 s, and 60º for 30 s. The percentage of deletion was obtained with the ddCT method, using healthy blood genomic control as calibrator.^11^

### Next‐generation sequencing

Identification of deletions in next‐generation sequencing data was performed in available urine and muscle in nine of the patients, as previously described.^12^ Libraries were prepared using the 16 kb PCR products and the Nextera DNA Flex Library Prep kit (Illumina), and samples were sequenced (2x150 bp) using the Illumina Nextseq 500 instrument. Raw sequencing reads were trimmed using Trimmomatic v0.39^13^ with options ILLUMINACLIP:illumina.fa:2:30:10 LEADING:3 TRAILING:3 SLIDINGWINDOW:4:15 MINLEN:36 and mapped against the complete human genome hg19 using bwa v0.7.15^14^ with default parameters. After removal of duplicates with SAMtools v1.9^15^, single‐nucleotide variants (SNVs) in mtDNA were identified and filtered for each individual sample using Genome Analysis Toolkit v3.8^16^ (indels were discarded and SNVs restricted to a quality score normalized by an allele depth of at least 2 and a maximum Fisher strand bias of 60). A new hg19 reference containing the alternate individual mtDNA SNVs was generated for each sample and reads were realigned with bwa. Reads with mapping quality below 30 and/or unmapped were filtered out using SAM tools. In order to identify deletions, alignments were analysed by Pindel v0.2.5^17^ with options –x 5 and –A 30. Only deletions identified between MT:1,000‐15,000 were kept for downstream analyses. To further reduce the false positive rate, *split reads* identified by Pindel as evidence for deletions were filtered based on a set of stringent criteria that had to be fulfilled by both the *split read* and its paired read: (1) median sequencing quality above 36, (2) no mismatches with the mtDNA sample‐specific reference, (3) mapping quality above 0, (4) aligned to the mtDNA chromosome with correct paired‐end orientation.

### Statistical analyses

The data were processed using SPSS v25 (IBM). A *P*‐value < 0.05 was considered statistically significant.

### Ethics

The study was approved by the Norwegian Regional Committee for Medical and Health Research Ethics (No: 2019/481), and written consent was obtained from all patients.

## Results

### Detection of single mtDNA deletions in urine sediment cell DNA

**Figure 1:**
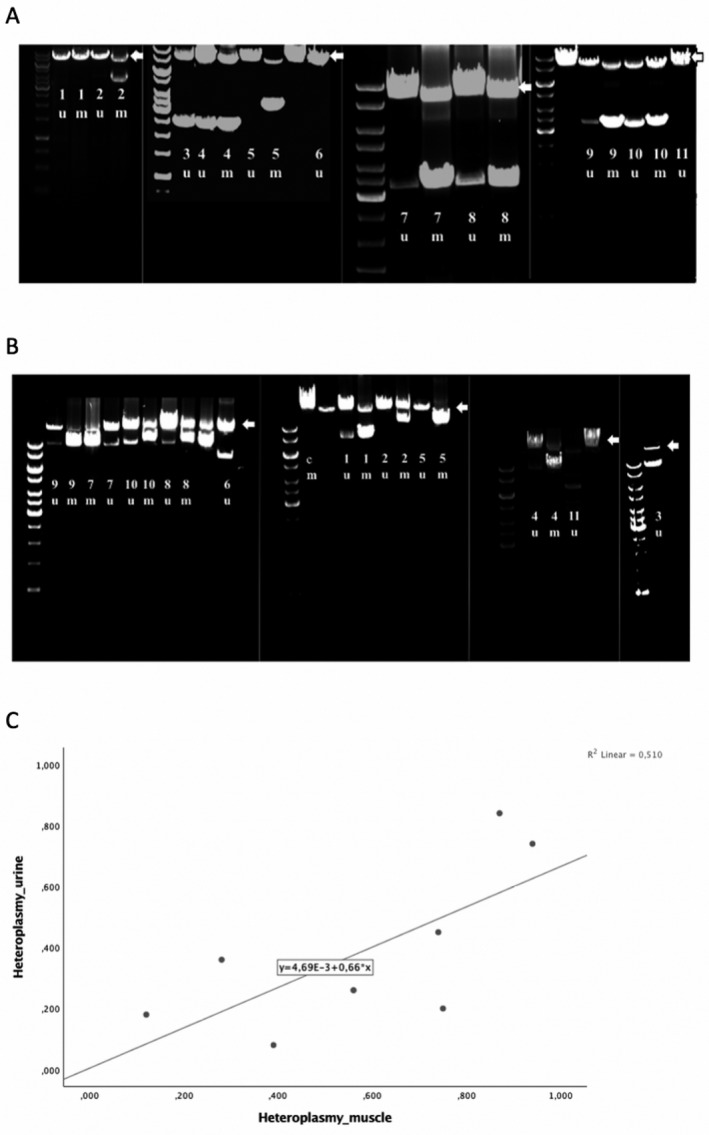
Long‐range PCR analysis in patients 1‐11 with single deletions in urinary sediment cells (u) and skeletal muscle (m) and control patient (c). The wild type amplicon is 8kb in Figure (A), and 16kb in Figure (B), and marked with an arrow. A 10 kb DNA ladder is shown to the left of each run, and vertical lines define separate experiments. In patient 6 and 11 only urinary samples were available. Due to technical problems with patient 3 muscle, we ran the urine sample alone, and used information concerning the deletion size obtained from the diagnostic assay. (C) shows a scatterplot illustrating the correlation between percent mutant mtDNA in muscle (x‐axis) and percent mutant mtDNA in urine (y‐axis) within the same individual. Heteroplasmy levels were determined using qPCR as described under methods.

The demographic and clinical data of the patients are summarized in Table 1. We detected a single deletion in urine samples from 9 of 11 patients (82%), either in 8 kb L‐PCR (Fig. 1A) and/or 16 kb L‐PCR (Fig. 1B). In the remaining two patients, the band was either very weak (pt 2), or undetectable (pt 5). Overloading the gel did not help with visualization.

**Table 1:** Details of phenotype, deletion size, and heteroplasmy level in adult patients investigated in this study. Deletion size in gel and NGS is based on both urine and muscle when available

Patient	Gender	Age	Phenotype	Detectable urine	Available muscle studied in parallel	Deletion size gel	Deletion size NGS	Heteroplasmy urine	Heteroplasmy muscle	Δ Heteroplasmy	Time (years) from muscle sampling to urinary sample
1	F	43	PEO	Yes	Yes	~ 7140 bp	7156 bp	84%	87%	3%	0
2	F	57	PEO	No	Yes	~ 4000 bp*	4407 bp	33%	49%	16%	0
3	F	54	PEO	Yes	Yes	~ 5‐6000 bp	Not performed	8%	39%	31%	0
4	F	17	PEO	Yes	Yes	~ 5500 bp	5800 bp	45%	74%	29%	0
5	M	54	PEO	No	Yes	~ 4500 bp*	4851 bp*	26%	56%	30%	13
6	F	59	PEO	Yes	No	~ 7390 bp	7386 bp	Not performed	Not available	Not available	18
7	F	56	PEO	Yes	Yes	~ 4500 bp	4977 bp	20%	75%	55%	0
8	F	30	PEO	Yes	Yes	~ 4500 bp	4977 bp	18%	12%	−6%	10
9	F	24	PEO	Yes	Yes	~ 4500 bp	Not performed	74%	94%	20%	0
10	M	32	PEO	Yes	Yes	~ 4500 bp	4977 bp	28%	36%	8%	1
11	F	22	PEO	Yes	No	~ 8140 bp	8284 bp	Not performed	12%	Not available	0

The size marked with asterix (*) indicates that it is not detectable in urine, and is therefore based on muscle findings.

F, female; M, male; NGS, next‐generation sequencing; PEO, progressive external ophthalmoplegia.

In six of the patients (pts 3, 4, 7‐10) the deletion was detected in both 8 kb and 16 kb L‐PCR. In three patients (pts 1, 6, 11), the deletion was only detectable on the 16 kb gel, indicating that these deletions most probably encompassed one of the 8 kb PCR primer sites. To confirm this, we used a process termed “walking”‐PCR, in which PCR primers are chosen to amplify regions adjacent to each of the original primers. Using this, we identified large deletions, each over 7 kb, in all three cases.

### Heteroplasmy levels and deletion size

Mean heteroplasmy level in urine was 38% ± 26 (range 8 ‐ 84%), and 57% ± 28 (range 12 – 94%) in muscle. The level was generally lower in urinary sediment cells, but we were able to define a correlation between muscle and urine levels within our cohort (*R* = 0.714, *P* = 0.031(Pearson correlation)) (Fig. 1C). This observation must be interpreted with caution, however, since the finding is based on nine patients only.

### Sequencing

In the two patients (pts 8 &10) in whom we performed Sanger sequencing; both had the common 4977 bp deletion. Pt 8 had deletion junction 8469: 13447. Pt 10 had deletion junction 8482: 13459, flanked with the 13 bp direct repeat ACCTCCCTCACCA; a known hotspot for deletion.

### Next‐generation sequencing

All deletions detected using L‐PCR in both muscle and urine were also detected in both muscle and urine using NGS (Table 1). However, we found that NGS generated evidence of additional deletions of varying read depth that were not found by L‐PCR.

## Discussion

The first aim of this study was to evaluate if urinary sediment cells offered a robust alternative to muscle biopsy for the diagnosis of single mtDNA deletions in adults. Our multicentre study shows that single deletions are detectable in urine in over 80% of the cases who had a clinical syndrome known to be caused by deletion, and in whom deletion was present in skeletal muscle.

We failed to detect deletions in two patients. Since we wanted to evaluate the efficacy of L‐PCR in the clinical setting, we did not use prior knowledge of deletion size in the skeletal muscle to generate shorter amplicons. The only common feature shared by these two individuals was deletion size; they had the smallest deletions in the cohort. Since their deletions were robustly detectable in skeletal muscle, one explanation for our failure to detect them in urinary cells is low heteroplasmy level. This, however, was not the case (Table 1). Skeletal muscle heteroplasmy levels in these two patients were not the lowest in the group (pt 2 = 49%, pt 5 = 56%; range 12–94%). Moreover, failure to detect small deletions would contradict previous studies that showed an inverse correlation between skeletal muscle heteroplasmy and deletion size ^7^, ^18^, ^19^.

Our second aim was to investigate whether heteroplasmy level in a patient´s urine was similar to that in skeletal muscle. We determined heteroplasmy level using qPCR. As clinical severity and prognosis in patients with mtDNA disorders are usually related to mutation load, the level of heteroplasmy is of clinical interest.^20^ As in previous studies,^10^ we found interpreting the level and impact of heteroplasmy in urinary cells problematical. Furthermore, four of the urine samples were taken at a later time point than the muscle biopsy and we know that this can affect the degree of heteroplasmy.^21^ Interestingly, in contrast with muscle, urinary epithelial cells are mitotic and thus capable of eliminating cells with comprised energy metabolism levels due to mutated mtDNA. That they retain mtDNA deletions at all is an interesting phenomenon, and our results suggest that they do this to levels that reflect what is found in postmitotic skeletal muscle.

Whether heteroplasmy levels, the deletion breakpoint and size are of clinical, predictive importance in patients with single deletions remains unclear. Various studies have shown contradictory findings, and we have summarized these in Table 2. Some studies have suggested that age of onset is the most important factor.^22^ More recently, Grady and colleagues reanalysed previously published data and found that there was indeed a predictive value of heteroplasmy, size, and site of deletion on disease burden and progression.^18^ Regardless of the debate concerning prognostic impact of estimating deletion size, site, and heteroplasmy level, we show that this information is readily available in urine.

**Table 2:** An overview over the diversity in previous studies on single deletions

Reference	Correlations	Correlations not found
Zeviani et al.^23^		No relationship between size and site of deletion and biochemistry or disease severity.
Holt et al.^24^		No correlation between heteroplasmy and clinical or biochemical severity No correlation between deletions site and phenotype
Aurè et al.^22^	More severe phenotype was associated with age of onset and the presence and proportion of deletion in blood associated with more severe phenotype (same nonsignificant trend was found in urine).	No correlation between phenotype and: ‐ Deletion size, ‐ Site of deletion ‐ Heteroplasmy
Yamashita et al.^25^	Deletion size correlated with: ‐ Age at onset (inverse) ‐ Phenotype (longer deletions in KSS) Number of deleted tRNAs correlated with: ‐ Age at onset (inverse) ‐ Phenotype (more in KSS) Correlation between site of deletion and age of onset	No correlation between heteroplasmy and: ‐ Age of onset ‐ Phenotype
Lopez‐Gallardo et al.^19^	Heteroplasmy correlated with: ‐ Age of onset (inverse) ‐ Deletion size (inverse) (only in CPEO patients) Inverse correlation between deletion size and age of onset Correlation between site of deletion and phenotype: ‐ Deletion involving the Mt‐CYB gene correlated with KSS phenotype	
Grady et al.^18^	Heteroplasmy correlates with: ‐ Deletion size (inverse) ‐ Phenotype ‐ Age of onset ‐ COX‐deficient fibre density Disease burden and progression is predicted by: ‐ Heteroplasmy ‐ Deletion size ‐ Site of deletion Deletion size correlates with: ‐ Location of deletion ‐ Age of onset	
Mancuso et al.^7^	Inverse correlation between heteroplasmy and: ‐ Deletion size ‐ Age at onset	No correlation between phenotype and: ‐ Heteroplasmy ‐ Deletion size Age not related to deletion size
Broomfield et al. ^9^	Childhood‐onset cohort. Weak correlation between heteroplasmy and age at onset	No correlation between age of onset and deletion size or site of deletion

Both L‐PCR and NGS detected mtDNA deletions in DNA extracted from urine, but we found that NGS generated evidence of additional deletions of varying read depth that were not found by L‐PCR. Since we used the same 16 kb amplicon used for L‐PCR, the noisier signal seen with NGS is possibly related to PCR amplification and may disappear when run on native DNA. The purpose of our study was not to perform a head‐to‐head comparison of these two methods, but to show that urine was a suitable tissue for diagnosis. Currently, we believe that L‐PCR is robust and straightforward enough for diagnostic purposes, however, future studies may well alter this conclusion, particularly if improvements in NGS techniques make it possible to sequence without prior amplification.

**Figure 2:**
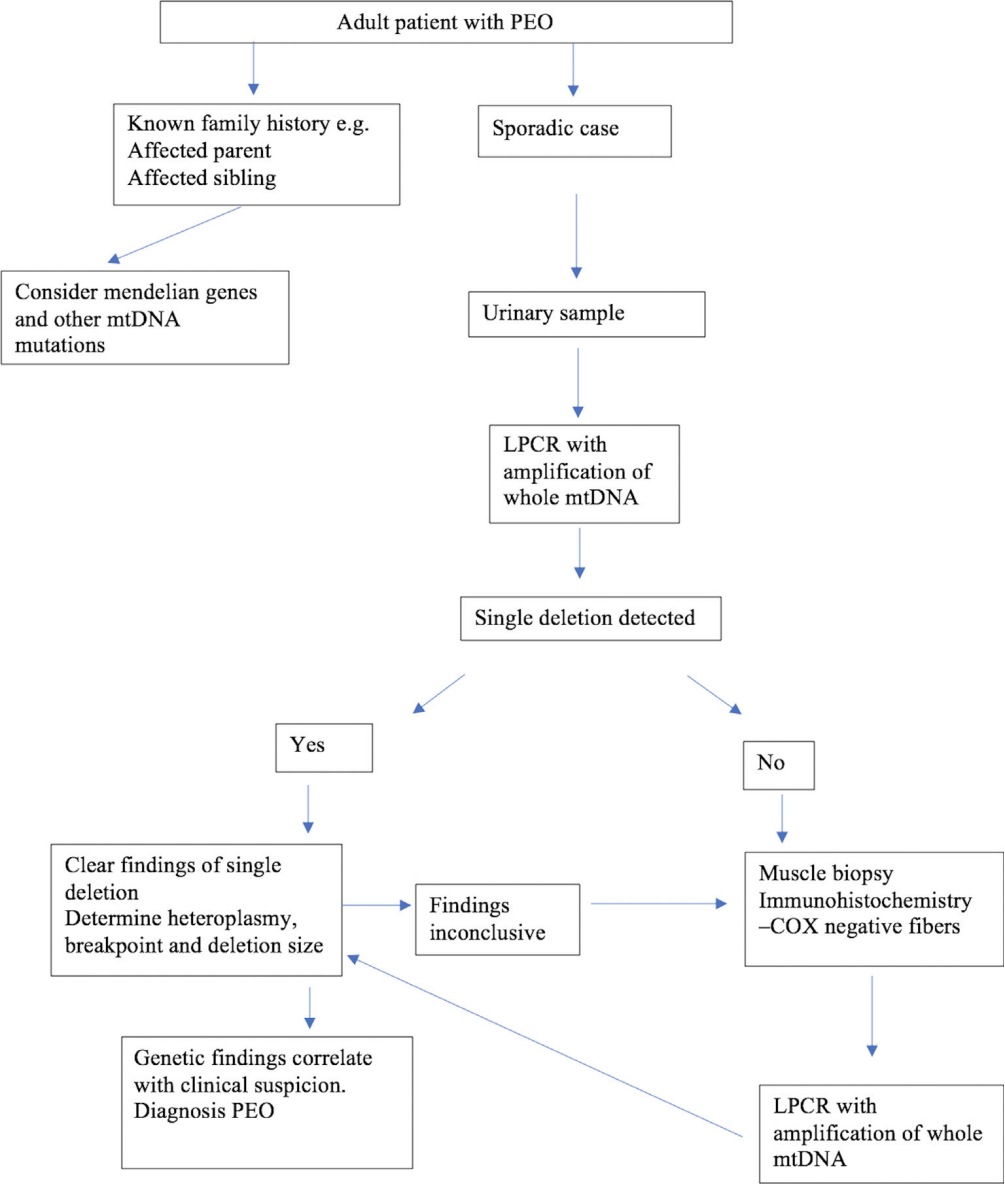
Proposed diagnostic algorithm in patients with progressive external ophthalmoplegia.

Our results suggest that urinary sediment cells are a viable alternative to muscle biopsy for the diagnosis of single mtDNA deletion disorder, even though we may miss the structural and pathological information provided by a muscle biopsy. Furthermore, although our study focused on adults with PEO, there is no reason to believe that diagnostic efficiency will be any less in children with KSS or Pearson syndrome. Our results show that urine provides evidence not only of the presence of a mtDNA deletion, but also permits the identification of breakpoints and the assessment of heteroplasmy. We would therefore recommend the following algorithm (Fig. 2): urine should be screened for single mtDNA deletions in patients with phenotypes known to be associated with this genetic defect. Muscle biopsy should be reserved for patients having high clinical suspicion but no detectable deletion in urine. We believe that this approach will reduce the need for muscle biopsy while maintaining diagnostic sensitivity.

## Author Contributions

KNV designed the research study, conducted the experiments, acquired, and analysed the data. LAB designed the research study and acquired data. GSN, CT, and PK conducted the NGS experiment and analysed the data. RdC, PI, and AS acquired data. All authors contributed to writing and editing of the manuscript.

## Conflict of Interest

The authors have declared that no conflict of interest exists.
